# Long-Term Preoperative Atorvastatin or Rosuvastatin Use in Adult Patients before CABG Does Not Increase Incidence of Postoperative Acute Kidney Injury: A Propensity Score-Matched Analysis

**DOI:** 10.3390/pathophysiology29030027

**Published:** 2022-07-11

**Authors:** Vladimir Shvartz, Eleonora Khugaeva, Yuri Kryukov, Maria Sokolskaya, Artak Ispiryan, Elena Shvartz, Andrey Petrosyan, Elizaveta Dorokhina, Leo Bockeria, Olga Bockeria

**Affiliations:** 1 Department of Surgical Treatment for Interactive Pathology, Bakulev Scientific Center for Cardiovascular Surgery, 121552 Moscow, Russia; doc.khugaeva@mail.ru (E.K.); masokolskaya@bakulev.ru (M.S.); ayispiryan@bakulev.ru (A.I.); adpetrosyan@bakulev.ru (A.P.); bockeria@bakulev.ru (L.B.); soleo2003@gmail.com (O.B.); 2 Department of Cardiovascular Surgery, Arrhythmology and Clinical Electrophysiology, Bakulev Scientific Center for Cardiovascular Surgery, 121552 Moscow, Russia; yurijkuban@mail.ru (Y.K.); dorokhinaelizaveta@yandex.ru (E.D.); 3 National Medical Research Center for Therapy and Preventive Medicine, 101990 Moscow, Russia; shvartz.en@ya.ru

**Keywords:** acute kidney injury, statins, coronary artery bypass graft, atorvastatin, rosuvastatin

## Abstract

Background: Acute kidney injury (AKI) is among the expected complications of cardiac surgery. Statins with pleiotropic anti-inflammatory and antioxidant effects may be effective in the prevention of AKI. However, the results of studies on the efficacy and safety of statins are varied and require further study. Methods: We conducted a retrospective cohort study to evaluate long-term preoperative intake of atorvastatin and rosuvastatin on the incidence of AKI, based on the “Kidney Disease: Improving Global Outcomes” (KDIGO) criteria in the early postoperative period after coronary artery bypass graft surgery (CABG). We performed propensity score matching to compare the findings in our study groups. The incidence of AKI was assessed on day 2 and day 4 after the surgery. Results: The analysis included 958 patients after CABG. After 1:1 individual matching, based on propensity score, the incidence of AKI was comparable both on day 2 after the surgery (7.4%) between the atorvastatin group and rosuvastatin group (6.5%) (OR: 1.182; 95%Cl 0.411–3.397; *p* = 0.794), and on postoperative day 4 between the atorvastatin group (3.7%) and the rosuvastatin group (4.6%) (OR: 0.723, 95%Cl 0.187–2.792; *p* = 0.739). Additionally, there were no statistically significant differences in terms of incidence of AKI after 1:1 individual matching, based on propensity score, between the rosuvastatin group and the control group both on postoperative day 2 (OR: 0.692; 95%Cl 0.252–1.899; *p* = 0.611) and day 4 (OR: 1.245; 95%Cl 0.525–2.953; *p* = 0.619); as well as between the atorvastatin group and the control group both on postoperative day 2 (OR: 0.549; 95%Cl 0.208–1.453; *p* = 0.240) and day 4 (OR: 0.580; 95%Cl 0.135–2.501; *p* = 0.497). Conclusion: Long-term statin use before CABG did not increase the incidence of postoperative AKI. Further, we revealed no difference in the incidence of post-CABG AKI between the atorvastatin and rosuvastatin groups.

## 1. Introduction

Acute kidney injury (AKI) is among the expected complications of cardiac surgery, associated with an increase in the incidence of subsequent progressive chronic kidney disease (CKD), as well as with high mortality, an increase in the length of patient stay duration in the hospital and the economic costs of patient treatment [1,2]. The incidence of AKI ranges from 10% to 70%, depending on cardiac surgery type and AKI definition employed [3]. Further, renal replacement therapy (RRT) is used in 1% of patients [4].

The mechanisms underlying AKI are multifactorial and are not yet fully understood. These include inflammation, oxidative stress, hypoperfusion, ischemic reperfusion injury of the renal tubules, erythrocyte hemolysis, neurohumoral activation, nephrotoxins, and mechanical factors [1,2,3]. Prevention of AKI is an important component of treating this patient category.

The 3-hydroxy-3-methylglutaryl-coenzyme A reductase inhibitors (statins) are drugs with proven efficacy (class I, level A) in patients with coronary artery disease. Their use by patients with known cardiovascular diseases (CVD) to prevent recurrent coronary or vascular events is almost not disputed [5,6]. The meta-analyses carried out proved the significant relative and absolute impact of statins on the primary prevention of most outcomes of CVD and mortality from all causes, while increasing the risk of some harmful consequences. Atorvastatin and rosuvastatin are the most effective, while atorvastatin has proven to be the safest statin [7].

In addition to their hypolipidemic effect, statins have been shown anti-inflammatory and pleiotropic effects. Statins can reduce the postoperative activation of white blood cells and endothelium and lead to a significant decrease in postoperative levels of proinflammatory cytokines in the blood serum [8,9]. One of the studies demonstrated that patients taking statins had lower levels of C-reactive protein, tumor necrosis factor-alpha (TNF-α), myeloperoxidase and several pro-inflammatory interleukins (IL) (IL-1, IL-6, IL-8); simultaneously, they had higher concentrations of anti-inflammatory IL-10 [10]. These findings allowed considering statins’ intake as one of the pharmacological approaches to AKI prevention.

In a number of non-randomized cohort studies, the use of statins in cardiac surgery patients has demonstrated a reduction in postoperative AKI and the need for subsequent RRT [11,12,13,14,15,16,17,18,19]. However, in another cohort study, statins did not have a significant effect on the incidence of AKI after cardiac surgery [19,20,21,22,23,24,25]; nor has the positive effect of statins on AKI been established in randomized clinical trials, although Zheng et al. noted an increased risk of postoperative AKI in patients receiving rosuvastatin [26,27,28,29,30].

Based on the results of meta-analyses of cohort studies, a reduction in the incidence of AKI was established in the statin group, compared with the control group [31,32]. On the contrary, in meta-analyses of randomized clinical trials, statin therapy was noted to increase the likelihood of postoperative AKI [33,34].

The European Association for Cardio-Thoracic Surgery (EACTS) Guidelines on Perioperative Medication in Adult Cardiac Surgery do not recommend initiating statin therapy shortly before cardiac surgery (Class III, Level A). They also emphasize that no data are available on whether patients already taking statins should continue or discontinue therapy preoperatively, although in common practice statins are continued perioperatively [35].

Accordingly, there are currently contradictory data on the effect of statins on kidney function in cardiac surgery. Initially considering statins as a preventive agent for the development of nephropathy after artificial circulation, they turned into “harmful” and “not recommended” medications. However, given the different research conditions, a heterogeneous population, and the different inclusion and exclusion criteria, not everything is so clear today. The hypothesis of our study is that long-term use of statins before heart surgery “does not increase the frequency of AKI.”

The objective of our study was to compare the effect of long-term preoperative intake of atorvastatin and rosuvastatin on the incidence of AKI in the early postoperative period after artery bypass graft surgery (CABG) surgery.

## 2. Materials and Methods

### 2.1. Study Population

We conducted a retrospective cohort study. The data were collected from the general electronic database of the «MedWork» clinic. This article includes an analysis of hospital stay in patients who underwent CABG from 2016 to 2020. Patients were selected from a single department: the Division of Surgical Treatment of Interactive Pathology. Overall, 1971 CABG operations were performed. Of those, all that were selected had met the inclusion/exclusion criteria.

The inclusion criteria were as follows:

- Performed CABG surgery—isolated or in combination with other interventions (correction of valvular pathology, correction of atrial fibrillation, etc.) in conditions of the cardiopulmonary bypass (CPB);
- Patients’ aged over 18 years old;
- Long-term (over 4 weeks) preoperative intake of atorvastatin or rosuvastatin and continued use of the same statin during hospital stay, or else—no pre/postoperative statin intake (control group).

The exclusion criteria were:

- CKD stages 4 and 5 patients;
- Some other statin intake (other than atorvastatin or rosuvastatin);
- Replacement of one statin for another before surgery or during hospital stay;
- The presence of concomitant cancer.

### 2.2. Data Collection

The standard preoperative examination involved collection of clinical and anamnestic data, along with laboratory and instrumental studies. Confirmation of concomitant comorbid pathology was performed by the specialists in the corresponding field (e.g., by an endocrinologist in case of diabetes mellitus, and a pulmonologist in case of chronic obstructive pulmonary disease (COPD)). Transthoracic echocardiography was performed in all patients before surgery, and during the early stages after surgery (on a daily basis, in order to assess hemodynamics, pericardial effusion, myocardial contractility, etc.). Laboratory diagnostics included an assessment of blood parameters both before surgery and for 6–7 days after. Electrocardiography (ECG) during hospital stay was performed in the first two days after surgery, using bedside monitors, then using a daily standard 12-channel ECG and Holter ECG monitoring according to indications, until the patient’s discharge. Intraoperative, intensive care unit (ICU), and early postoperative data were collected from the general electronic database of the «MedWork» clinic in compliance with all legal principles.

This study protocol complied with the ethical guidelines of the 1975 Declaration of Helsinki, as well as with the Ethical Guidelines for Epidemiological Research by the Government of the Russian Federation. This study was approved by the Ethics Committee at Bakulev Center for Cardiovascular Surgery (Protocol #2 date 27 January 2021). Written informed consent was obtained from every patient before each surgery by the physicians.

### 2.3. Study Groups

For the final analysis, we selected 958 anamneses of post-CABG patients meeting all inclusion and exclusion criteria.

The atorvastatin group consisted of 164 patients with a long-term preoperative and early postoperative period of atorvastatin intake. The rosuvastatin group included 296 patients and was based on similar principles. The control group comprised 498 patients not taking statins both before and after the surgery.

### 2.4. Endpoints

The primary clinical endpoint was the development of AKI. The presence of AKI was identified sensu the KDIGO criteria: occurrence of AKI was defined as either an increase in serum creatinine ≥0.3 mg/dL (≥26.5 mmol/L) within 48 h, or an increase up to ≥1.5-fold the baseline value within 7 days) [36].

The incidence of AKI was assessed on days 2 and 4 after the operation. Accordingly, we distinguished an early AKI (AKI 1) on postoperative day 2 and a delayed AKI (AKI 2) emerging between days 2 and 4 after the surgery.

The secondary endpoints were the length of hospital stay and hospital mortality. The analysis of surrogate data included determining the frequencies of fluid in the pericardium, fluid in the pleura, dynamics of laboratory parameters (white blood cells, neutrophils, AST, ALT, etc.).

### 2.5. Surgery

The CABG operation was performed on a beating heart under CPB conditions. Traditionally, the left internal thoracic artery with bypass grafting of the anterior interventricular artery, along with the great saphenous vein (GSV), were used as conduits for bypassing the rest of the coronary arteries. In cases of conduit deficiency (history of phlebectomy, GSV varicose), the radial artery was used. In cases of combined CABG with aortic valve replacement, the first step was to collect the planned number of conduits, after which the aortic valve replacement was performed. Then, after restoring the integrity of aorta and right atrium, the patient’s body was subjected to warming up to 36.6 °C, and the cardiac activity was restored. Next, myocardial revascularization was conducted on a beating heart under CPB conditions. Shunting of the target coronary arteries was carried out via imposing a distal anastomosis, and then proximal anastomoses were formed on parietal squeezed aorta. The quality of formed anastomoses was assessed using intraoperative bypass angiography, which enabled identification and elimination of their defects in a timely manner.

### 2.6. Statistical Analysis

Statistical data processing was carried out using the SPSS^®^ Statistics 28.0 software (IBM, Armonk, NY, USA). The primary database elements were represented by quantitative and categorical variables. All quantitative data were examined for normality using the Shapiro–Wilk test. With a normal distribution, the data were shown as the mean and standard deviation (M ± SD), while with a non-normal distribution, they were presented as a median and interquartile range: Me (Q1; Q3). To compare two independent samples, we employed the parametric Student’s *t*-test or the non-parametric Mann–Whitney U test for quantitative variables, and Pearson’s chi-squared test or Fisher’s exact test for categorical variables. Intergroup differences were considered statistically significant at *p* < 0.05.

Propensity score matching was performed to compare the findings in our study groups. A total of 958 patients were matched in a 1:1 ratio based on propensity scores. Propensity scores were calculated for each patient using multivariate logistic regression based on the following covariates: age, gender, weight, diabetes, COPD, hypertension, number of distal anastomoses, medicamentous therapy (beta blockers, angiotensin-converting-enzyme (ACE) inhibitors, non-steroidal anti-inflammatory drugs (NSAIDs), both at baseline and in the early postoperative period, and using calipers of width equal to 0.001 of the standard deviation of the propensity score logit.

## 3. Results

### 3.1. Atorvastatin vs. Rosuvastatin

The characteristics of the entire group and the propensity score-matched populations are shown in Table 1.

Of the enrolled 958 patients, the analysis included 164 patients taking atorvastatin and 296 patients taking rosuvastatin. After 1:1 individual matching, based on propensity score, 108 pairs were generated. The incidence of AKI in both groups was comparable both on postoperative days 2 (7.4% in the atorvastatin group vs. 6.5% in the rosuvastatin group; OR: 1.182; 95%Cl 0.411–3.397; *p* = 0.794), and 4 (3.7% in the atorvastatin group vs. 4.6% in the rosuvastatin group; OR: 0.723, 95%Cl 0.187–2.792; *p* = 0.739) (Table 2).

Dynamics of echocardiography parameters, laboratory data, and medicaments therapy are presented in Tables S1 and S4, Supplementary Materials.

### 3.2. The Rosuvastatin Group vs. the Control Group

Of 958 enrolled patients, 296 subjects were exposed, and 498 were not exposed, to long-term preoperative rosuvastatin intake. After 1:1 individual matching, based on propensity score, 223 pairs were generated (Table 3). The incidence of AKI did not differ significantly between the rosuvastatin and control groups both on postoperative days 2 (3.1 and 4%, correspondingly; OR: 0.692; 95%Cl 0.252–1.899; *p* = 0.611) and 4 (OR: 1.245; 95%Cl 0.525–2.953; *p* = 0.619) (Table 2).

Dynamics of echocardiography parameters, laboratory data, along with medicamentous therapy are presented in Tables S2 and S5, Supplementary Materials.

### 3.3. The Atorvastatin Group vs. the Control Group

Of the enrolled 958 patients, 164 were exposed and 498 were not exposed to preoperative atorvastatin therapy. After 1:1 individual matching, based on propensity score, 124 pairs were generated (Table 4). There were no significant differences in the values of baseline characteristics between the atorvastatin and control groups in the propensity score-matched populations. AKI on postoperative day 2 was detected in 7 (5.6%) patients in the atorvastatin group, and in 12 (9.7%) patients in the control group, but the differences between the groups had no statistical significance (OR: 0.549; 95%Cl 0.208–1.453; *p* = 0.240). There were also no differences between the groups in the incidence of AKI on postoperative day 4 (2.4% and 4%, respectively; OR: 0.580; 95%Cl 0.135–2.501, *p* = 0.497) (Table 2).

Dynamics of echocardiography parameters, laboratory data, along with medicamentous therapy are presented in Tables S3 and S6, Supplementary Materials.

## 4. Discussion

A number of risk factors for AKI have been identified in retrospective cohort studies: they can be classified into preoperative, intraoperative and postoperative. The former category includes female gender, older age, multiple comorbidities, and obesity [37]. Intraoperative risk factors comprise duration of aortic cross-clamping, duration of CPB, non-pulsatile flow, hypothermic CPB, repeated and emergency open cardiac surgery, along with interventions on heart valves [38,39]. The latter category encompasses exposure to nephrotoxins, postoperative development of cardiogenic shock, low hematocrit, sepsis, etc. [40,41].

One of probable pharmacological approaches to the prevention of AKI is the use of statins. Some studies have demonstrated a renoprotective role of statins based on their pleiotropic anti-inflammatory and antioxidant effects [8,9,11,12,13,14,15,16,17,18,32,33]. However, in our study, statin use did not have a significant positive effect on the incidence of AKI after CABG, compared with the control group, but also did not increase the risks of the incidence of AKI, which was consistent with some cohort and randomized clinical trials [19,20,21,22,23,24,25,26,27,28,29,42].

It should be noted that several studies suggested that statin therapy increased the likelihood of AKI, with one meta-analysis finding that rosuvastatin had a higher risk of postoperative AKI, compared with atorvastatin [30,33,34]. The authors of these studies associated the increase in the incidence of AKI against the background of the intake of statins with the fact that ischemic damage to the liver and kidneys occurred against the background of cardiac surgery. Statins are mainly metabolized by liver and kidney, which could aggravate the stress on these organs and increase the incidence of AKI. From the pathophysiological point of view the renal toxicity is also mediated by activation of bone marrow cells, endothelial cells and kidney epithelial cells caused by cardiopulmonary bypass, which leads to the formation of reactive oxygen species and the release of inflammatory mediators. Pro-inflammatory cytokines secreted by infiltrating and resident cells contribute to further tissue damage until the inflammation resolves and proliferation of the tubular epithelium occurs, leading to the return of normal tissue function [43].

The difference between atorvastatin and rosuvastatin in the incidence of AKI is explained, first of all, by the lipophilicity of the former and the hydrophilicity of the latter. According to the literature, the pleiotropic effect differs in individual statins, and is more pronounced in lipophilic statins [44]. Additionally, because only 2% of atorvastatin metabolites are excreted by the kidneys, in contrast to rosuvastatin (10%). This fact is an additional argument in favor of the atorvastatin intake in patients with kidney pathology. Atorvastatin is positioned as a ‘renal’ statin in the NICE Guidelines [45].

In our study, statin use was found to be safe, and no difference in incidence of AKI was established between atorvastatin and rosuvastatin. Such inconsistent results were likely due to varying levels of cardiac surgery complexity, timing of statin intake commencement (pre- or postoperative statin intake commencement), duration of statin use, and the lack of a single definition of AKI across various studies.

There are standardized diagnostic criteria for AKI developed worldwide: RIFLE (2004), AKIN (2007) and KDIGO (2012). The latter is a combination of AKIN and RIFLE criteria; hence it demonstrates greater sensitivity in the diagnosis of AKI [36,46,47].

An important factor, in our opinion, is the duration of statin administration required to achieve the maximum pleiotropic effect. Although statins, in particular atorvastatin, have been shown to have a rapid antioxidant effect in patients who underwent scheduled CABG [48], other studies demonstrated that it usually takes 14 days or more of statin therapy to achieve a full anti-inflammatory effect [49,50]. This finding was supported by meta-regression analysis, in which statins were associated with a 3% daily reduction in the risk of postoperative atrial fibrillation, highlighting the added benefit of earlier commencement of preoperative therapy [51].

At present, the significance of the starting time of taking statins and the duration of their intake on the incidence of AKI after cardiac surgery has not yet been confirmed, since there are not enough randomized clinical trials that would evaluate the incidence of AKI, specifically, after the long-term preoperative statin intake. However, in all of these studies, AKI was identified without using RIFLE, AKIN, or KDIGO criteria, preoperative statin use was limited to 3–4 weeks, and no postoperative statin intake took place [27,52,53]. In our study, AKI determination was based on KDIGO criteria, preoperative statin intake was over 4 weeks, and it was continued during hospital stay.

### Limitations of This Study

This analysis had some drawbacks related to the retrospective approach.

First of all, despite the fact that all data were collected from the general electronic database of our «Medwork» clinic with the standard mandatory data entry, the retrospective analysis did not exclude partial data loss.

Second, the use of propensity score matching did not completely rule out the latent bias that could affect the results.

Finally, this study did not take into account the possible different doses of statins, both preoperatively and in the early postoperative period.

## 5. Conclusions

In this study, we conducted a comparative analysis of the effect of long-term preoperative intake of atorvastatin and rosuvastatin on the development of AKI in the early postoperative period after CABG. Our findings revealed that long-term use of either of the two statins did not increase the incidence of postoperative AKI after CABG, compared with the control group. Further, we found no difference in the incidence of AKI between the atorvastatin and rosuvastatin groups.

## Figures and Tables

**Table 1:** Clinical data and operative data.

Parameters	Unmatched Raw Data			Propensity Matched 1:1		
	Atorvastatin (*n* = 164)	Rosuvastatin (*n* = 296)	*p*	Atorvastatin (*n* = 108)	Rosuvastatin (*n* = 108)	*p*
Age, years	63 (56–67)	62 (57–67)	0.887	64 (57.5–68)	63 (58.5–68)	0.763
Male, *n* (%)	125 (76.2)	229 (77.4)	0.780	78 (72.2)	83 (76.9)	0.435
Weit, kg	85 (76–95)	84 (75–92)	0.270	82 (75–91.5)	82.5 (74.5–92)	0.676
BMI	29.2 ± 3.9	29 ± 3.8	0.594	28.9 ± 3.9	29.2 ± 3.7	0.528
Angina 3–4 class, *n* (%)	129 (78.7)	211 (71.3)	0.084	83 (76.9)	78 (72.2)	0.435
Diabetes, *n* (%)	26 (15.9)	70 (23.6)	0.049	22 (20.4)	16 (14.8)	0.284
Lung disease, *n* (%)	31 (18.9)	26 (8.8)	0.002	15 (13.9)	21 (19.4)	0.273
Hypertension, *n* (%)	158 (96.3)	278 (93.9)	0.381	103 (95.4)	104 (96.3)	1.000
Previous MI, *n* (%)	93 (56.7)	166 (56.1)	0.897	59 (54.6)	62 (57.4)	0.681
Previous stroke/TIA, *n* (%)	6 (3.7)	8 (2.7)	0.580	6 (5.6)	2 (1.9)	0.280
Previous AF, *n* (%)	15 (9.1)	32 (10.8)	0.572	11 (10.2)	15 (13.9)	0.403
Smoker, *n* (%)	52 (31.7)	75 (25.3)	0.149	30 (27.8)	33 (30.6)	0.653
Bypass time, min	88 (63.5–122)	89 (67–116)	0.889	90 (70–127.5)	94 (72–117)	0.829
Cardioplegia, *n* (%)	18 (11)	41 (13.9)	0.377	13 (12)	16 (14.8)	0.549
Cross-clamp time, min	69.2 ± 23.8	63.6 ± 20.9	0.379	75.8 ± 21.5	62.4 ± 12.5	0.064
AV Replacement, *n* (%)	12 (7.3)	22 (7.4)	0.964	11 (10.2)	6 (5.6)	0.312
MV Replacement, *n* (%)	4 (2.4)	10 (3.4)	0.778	3 (2.8)	6 (5.6)	0.498
DCA, *n*	1 (1–2)	2 (1–2)	<0.001	1 (1–2)	2 (1–2)	0.102
DCA 1, *n* (%)	100 (61)	91 (30.7)	<0.001	61 (56.5)	44 (40.7)	0.021
DCA 2, *n* (%)	45 (27.4)	160 (54.1)	<0.001	37 (34.3)	61 (56.5)	0.001
DCA 3 and more, *n* (%)	10 (6.1)	24 (8.1)	0.401	10 (9.3)	3 (2.8)	0.083
LV time, hours	10 (7–17.3)	12 (8–17)	0.100	10 (7.5–16.7)	12.6 (9–19.6)	0.072

BMI—body mass index, AV—aortic valve, MV—mitral valve, DCA—distal coronary anastomoses, and LV—lung ventilation.

**Table 2:** Clinical data and operative data.

**Parameters**	**Unmatched Raw Data**					**Propensity Matched 1:1**				
	**Atorvastatin** **(*n* = 164)**	**Rosuvastatin** **(*n* = 296)**	**OR**	**95% Cl**	***p***	**Atorvastatin** **(*n* = 108)**	**Rosuvastatin** **(*n* = 108)**	**OR**	**95% Cl**	***p***
AKI 1, *n* (%)	11 (6.7)	15 (5.1)	1.363	0.6–3.1	0.526	8 (7.4)	7 (6.5)	1.182	0.4–3.4	0.794
AKI 2, *n* (%)	8 (4.9)	17 (5.7)	0.657	0.3–1.6	0.343	4 (3.7)	5 (4.6)	0.723	0.2–2.8	0.739
LOS, days	7 (6–9)	7 (6–8)			0.039	7 (6–10)	7 (6–9)			0.676
Hospital mortality, *n* (%)	1 (0.6)	2 (0.7)	0.902	0.1–10.0	1.000	1 (0.9)	0 (0)			1.000
**Parameters**	**Unmatched Raw Data**					**Propensity Matched 1:1**				
	**Rosuvastatin** **(*n* = 296)**	**No Statins** **(*n* = 498)**	**OR**	**95% Cl**	***p***	**Rosuvastatin** **(*n* = 245)**	**No Statins** **(*n* = 245)**	**OR**	**95% Cl**	***p***
AKI 1, *n* (%)	15 (5.1)	28 (5.6)	0.751	0.4–1.4	0.385	7 (3.1)	9 (4)	0.692	0.3–1.9	0.611
AKI 2, *n* (%)	17 (5.7)	22 (4.4)	1.001	0.5–1.9	0.997	13 (5.8)	10 (4.5)	1.245	0.5–2.9	0.619
LOS, days	7 (6–8)	6 (6–7)			<0.001	7 (6–7)	6 (6–7)			0.077
Hospital mortality, *n* (%)	2 (0.7)	2 (0.4)	1.687	0.2–12.0	0.632	2 (0.9)	1 (0.4)	2.009	0.2–22.3	1.000
**Parameters**	**Unmatched Raw Data**					**Propensity Matched 1:1**				
	**Atorvastatin** **(*n* = 164)**	**No Statins** **(*n* = 498)**	**OR**	**95% Cl**	***p***	**Atorvastatin** **(*n* = 124)**	**No Statins** **(*n* = 124)**	**OR**	**95% Cl**	***p***
AKI 1, *n* (%)	11 (6.7)	28 (5.6)	1.024	0.5–2.1	0.948	7 (5.6)	12 (9.7)	0.549	0.2–1.4	0.240
AKI 2, *n* (%)	8 (4.9)	22 (4.4)	0.658	0.3–1.5	0.326	3 (2.4)	5 (4)	0.580	0.1–2.5	0.497
LOS, days	7 (6–9)	6 (6–7)			<0.001					0.368
Hospital mortality, *n* (%)	1 (0.6)	2 (0.4)	1.521	0.12–16.9	0.731	1 (0.8)	1 (0.8)	1.000	0.1–16.2	1.000

AKI—acute kidney injury; LOS—length of stay.

**Table 3:** Clinical data and operative data.

Parameters	Unmatched Raw Data			Propensity Matched 1:1		
	Rosuvastatin (*n* = 296)	No Statins (*n* = 498)	*p*	Rosuvastatin (*n* = 223)	No Statins (*n* = 223)	*p*
Age, years	62 (57–67)	62 (56–67)	0.655	62 (57–67)	63 (57–68)	0.311
Male, *n* (%)	229 (77.4)	399 (80.1)	0.356	183 (82.1)	184 (82.5)	0.901
Weit, kg	84 (75–92)	83 (75–94)	0.731	84 (75.5–92)	82 (75–93)	0.489
BMI	29 ± 3.8	28.7 ± 3.9	0.365	28.9 ± 3.8	28.6 ± 3.8	0.348
Angina 3–4 class, *n* (%)	211 (71.3)	370 (74.3)	0.354	160 (71.7)	175 (78.5)	0.100
Diabetes, *n* (%)	70 (23.6)	105 (21.1)	0.408	50 (22.4)	53 (23.8)	0.736
Lung disease, *n* (%)	26 (8.8)	42 (84)	0.865	18 (8.1)	18 (8.1)	1.000
Hypertension, *n* (%)	278 (93.9)	455 (91.4)	0.191	209 (93.7)	211 (94.6)	0.686
Previous MI, *n* (%)	166 (56.1)	266 (53.4)	0.435	128 (57.4)	121 (54.3)	0.504
Previous stroke/TIA, *n* (%)	8 (2.7)	24 (4.8)	0.143	7 (3.1)	8 (3.6)	1.000
Previous AF, *n* (%)	32 (10.8)	29 (5.8)	0.011	20 (9)	18 (8.1)	0.734
Smoker, *n* (%)	75 (25.3)	103 (20.7)	0.126	59 (26.5)	41 (18.4)	0.041
Bypass time, min	89 (67–116)	83 (60–112)	0.025	85 (68–113.5)	88 (65–115.5)	0.805
Cardioplegia, *n* (%)	41 (13.9)	43 (8.6)	0.023	25 (11.2)	26 (11.7)	0.882
Cross-clamp time, min	63.6 ± 20.9	71.9 ± 21.7	0.083	63.2 ± 15.8	70.7 ± 20.8	0.167
AV Replacement, *n* (%)	22 (7.4)	27 (5.4)	0.255	12 (5.4)	16 (7.2)	0.435
MV Replacement, *n* (%)	10 (3.4)	12 (2.4)	0.503	5 (2.2)	7 (3.1)	0.771
DCA, *n*	2 (1–2)	2 (1–2)	0.007	2 (1–2)	2 (1–2)	0.828
DCA 1, *n* (%)	91 (30.7)	183 (36.7)	0.085	77 (34.5)	76 (34.1)	0.921
DCA 2, *n* (%)	160 (54.1)	254 (51)	0.405	132 (59.2)	138 (61.9)	0.561
DCA 3 and more, *n* (%)	24 (8.1)	15 (3)	0.002	14 (6.3)	9 (4)	0.284
LV time, hours	12 (8–17)	13 (8–20)	0.172	12 (8–17)	13 (9–19)	0.111

BMI—body mass index, AV—aortic valve, MV—mitral valve, DCA—distal coronary anastomoses, and LV—lung ventilation.

**Table 4:** Clinical data and operative data.

Parameters	Unmatched Raw Data			Propensity Matched 1:1		
	Atorvastatin (*n* = 164)	No Statins (*n* = 498)	*p*	Atorvastatin (*n* = 124)	No Statins (*n* = 124)	*p*
Age, years	63 (56–67)	62 (56–67)	0.793	61.3 ± 7.5	62.2 ± 8.5	0.407
Male, *n* (%)	125 (76.2)	399 (80.1)	0.286	102 (82.3)	97 (78.2)	0.425
Weit, kg	85 (76–95)	83 (75–94)	0.164	83.5 (76–94.5)	84 (75.5–95)	0.707
BMI	29.2 ± 3.9	28.7 ± 3.9	0.191	29 ± 4	29 ± 4.2	0.992
Angina 3–4 class, *n* (%)	129 (78.7)	370 (74.3)	0.261	94 (75.8)	99 (79.8)	0.445
Diabetes, *n* (%)	26 (15.9)	105 (21.1)	0.142	23 (18.5)	25 (20.2)	0.748
Lung disease, *n* (%)	31 (18.9)	42 (8.4)	<0.001	22 (17.7)	23 (18.5)	0.869
Hypertension, *n* (%)	158 (96.3)	455 (91.4)	0.035	118 (95.2)	120 (96.8)	0.749
Previous MI, *n* (%)	93 (56.7)	266 (53.4)	0.463	68 (54.8)	61 (49.2)	0.374
Previous stroke/TIA, *n* (%)	6 (3.7)	24 (4.8)	0.535	6 (4.8)	5 (4)	1.000
Previous AF, *n* (%)	15 (9.1)	29 (5.8)	0.138	11 (8.9)	8 (6.5)	0.634
Smoker, *n* (%)	52 (31.7)	103 (20.7)	0.004	40 (32.3)	31 (25)	0.221
Bypass time, min	88 (63.5–122)	83 (60–112)	0.065	90 (68.5–125)	86 (65–116)	0.347
Cardioplegia, *n* (%)	18 (11)	43 (8.6)	0.381	12 (9.7)	16 (12.9)	0.409
Cross-clamp time, min	69.2 ± 23.8	71.9 ± 21.7	0.675	71.5 ± 21.6	74.2 ± 23.4	0.758
AV Replacement, *n* (%)	12 (7.3)	27 (5.4)	0.444	9 (7.3)	10 (8.1)	1.000
MV Replacement, *n* (%)	4 (2.4)	12 (2.4)	1.000	2 (1.6)	4 (3.2)	0.684
DCA, *n*	1 (1–2)	2 (1–2)	<0.001	1 (1–2)	1 (1–2)	0.364
DCA 1, *n* (%)	100 (61)	183 (36.7)	<0.001	80 (64.5)	71 (57.3)	0.242
DCA 2, *n* (%)	45 (27.4)	254 (51)	<0.001	37 (29.8)	50 (40.3)	0.084
DCA 3 and more, *n* (%)	10 (6.1)	15 (3)	0.102	7 (5.6)	3 (2.4)	0.334
LV time, hours	10 (7–17.3)	13 (8–20)	0.005	9.7 (6.6–16.8)	12 (8–17)	0.040

BMI—body mass index, AV—aortic valve, MV—mitral valve, DCA—distal coronary anastomoses, and LV—lung ventilation.

## Data Availability

The datasets analyzed during the current study are publicly available. The data are available in the general repository “Open Science Framework” at the link https://doi.org/10.17605/OSF.IO/NT93Y.

## Supplementary Materials

The following supporting information can be downloaded at: https://www.mdpi.com/article/10.3390/pathophysiology29030027/s1, Table S1: Dynamics of echocardiography parameters and laboratory data (Atorvastatin vs. Rosuvastatin); Table S2: Dynamics of echocardiography parameters and laboratory data (Rosuvastatin vs. Control Group); Table S3: Dynamics of echocardiography parameters and laboratory data (Atorvastatin vs. Control Group); Table S4: Medicamentous therapy (Atorvastatin vs. Rosuvastatin); Table S5: Medicamentous therapy (Rosuvastatin vs. Control Group); Table S6: Medicamentous therapy (Atorvastatin vs. Control Group).
