# Supplementary material for: Long-Term Preoperative Atorvastatin or Rosuvastatin Use in Adult Patients before CABG Does Not Increase Incidence of Postoperative Acute Kidney Injury: A Propensity Score-Matched Analysis
Source: Pathophysiology. 2022 Jul 11;29(3):354–64. doi: 10.3390/pathophysiology29030027 (PMC9326680; doi:10.3390/pathophysiology29030027)
Supplement: Supplementary file 1 [file pathophysiology-29-00027-s001.zip › Tables S1-S6.pdf]

Table S1. Dynamics of echocardiography parameters and laboratory data (Atorvastatin vs. Rosuvastatin).

Parameter	Unmatched raw data			Propensity matched 1:1		
	Atorvastatin (n=164)	Rosuvastatin (n=296)	p	Atorvastatin (n=108)	Rosuvastatin (n=108)	p
Echocardiographic indicators initially						
LVEF, %	59.5 (54.2-63)	59 (55.7-62.1)	0.731	59 (55-63)	59 (55.1-61.8)	0.773
LVESD, cm	3.4 (3.1-3.7)	3.3 (3.1-3.6)	0.307	3.4 (3.1-3.7)	3.3 (3.1-3.6)	0.630
LVEDD, cm	5 (4.7-5.4)	5 (4.7-5.3)	0.129	5 (4.7-5.3)	5 (4.7-5.2)	0.494
LVESV, ml	50.5 (39.5-60)	47.2 (37.9-58.1)	0.202	47.4 (39-58.1)	45.5 (38-57.5)	0.564
LVEDV, ml	123.8 (105.5-141.3)	118.2 (102.4-135.3)	0.043	118.2 (101.2-136.2)	118.2 (102.4-129.5)	0.361
Echocardiographic indicators 2 day after operation						
LVEF, %	55 (52-56)	55.7 (53-57)	0.010	55 (52-56)	55.2 (54-56.8)	0.054
LVESV, ml	41 (34-48)	43 (35-50)	0.299	40 (33-48)	42.5 (35-50.9)	0.274
LVEDV, ml	95 (77.5-114)	97.3 (81-112)	0.683	91.5 (74-108)	93.2 (83-112)	0.441
Fluid in the pericardium, n (%)	16 (9.8)	25 (8.4)	0.577	13 (12)	8 (7.4)	0.173
Fluid in the pleura, n (%)	25 (15.2)	46 (15.5)	0.314	20 (18.5)	19 (17.6)	0.267
Echocardiographic indicators 4 day after operation						
LVEF, %	55 (53-56.7)	55.9 (54-57)	0.055	55 (53-56.9)	55.9 (54.4-57)	0.062
LVESV, ml	41 (34-50)	43 (35-50.9)	0.409	40 (34-47.4)	44.1 (37.9-50.9)	0.069
LVEDV, ml	97.5 (80.5-114)	97 (82-113)	0.824	95.5±20.9	99.9±22.7	0.193
Fluid in the pericardium, n (%)	40 (24.4)	46 (15.5)	0.029	28 (25.9)	22 (20.4)	0.233
Fluid in the pleura, n (%)	37 (22.6)	37 (12.5)	0.001	29 (26.9)	16 (14.8)	0.003
Laboratory characteristics initially						
WBC, 10*9/l	7.7 (6.6-9)	7.7 (6.6-8.9)	0.982	7.6 (6.5-9)	7.7 (6.8-9)	0.634
Neutrophils, 10*9/l	4.6 (3.9-5.6)	4.6 (3.8-5.4)	0.800	4.6 (3.7-5.4)	4.6 (3.9-5.3)	0.918
Neutrophils, %	58.7 (53-64.8)	58.4 (52.2-64.6)	0.844	60 (53.5-66)	58.2 (52.2-65.7)	0.485
Creatinine, mmol/l	86.3 (75.8-96.2)	85.3 (74.5-99.7)	0.527	85 (75.8-96.2)	85 (76.5-98.1)	0.574
Glucose, mmol/l	5.6 (5-6.4)	5.5 (6-6.3)	0.860	5.6 (5-6.4)	5.5 (5-6.1)	0.595
AAT	23 (18-38)	22 (18.5-28.5)	0.653	22 (17-36)	23 (19-35.5)	0.466
ALT	27 (18-40)	26.5 (19-41.5)	0.786	24.5 (17-38)	25.5 (19-39)	0.444
Potassium, mmol/l	4.4 (4.2-4.7)	4.5 (4.3-4.9)	0.138	4.4 (4.3-4.7)	4.5 (4.2-4.9)	0.226
Laboratory characteristics 2 day after operation						
WBC, 10*9/l	12.8 (10.5-15)	13 (10.7-16.3)	0.391	13 (10.7-16)	13.6 (10.8-16.6)	0.702
Neutrophils, 10*9/l	10.9 (8.8-13.7)	11.2 (8.9-15.4)	0.364	11.4±3.7	12.7±4.3	0.094
Neutrophils, %	83.4 (76.9-86.7)	83.2 (76.7-87.4)	0.773	83.2 (76.9-87.8)	84.4 (80.3-87.9)	0.575
Creatinine, mmol/l	80.2 (69.1-97.9)	80.1 (68-94.7)	0.773	81.5 (70.9-95)	81 (69.1-98.2)	0.869
Glucose, mmol/l	7.3 (6-9.3)	7.6 (6.4-10)	0.051	8 (6.3-9.7)	7.7 (6.7-10.2)	0.409
AAT	33 (23.5-60.5)	31 (22-49)	0.202	34 (24-65)	32 (23-50)	0.435
ALT	24 (16.5-37.5)	22 (16-32)	0.171	23.5 (15-37)	22 (15-31)	0.395

Potassium, mmol/l	4.1 (3.8-4.5)	4.2 (3.9-4.5)	0.241	4.2±0.4	4.2±0.5	0.915
Laboratory characteristics 4 day after operation						
WBC, 10*9/l	10.5 (9-13.2)	11.3 (9.4-13.6)	0.101	10.6 (9-13.1)	11.1 (9.4-13.2)	0.446
Neutrophils, 10*9/l	7.8 (6.2-10)	7.5 (5.9-9.9)	0.547	8 (6.2-10.2)	7.5 (6.5-9.8)	0.884
Neutrophils, %	65.7 (60.3-71.9)	66 (59.8-75.2)	0.708	66.1 (61.4-72.8)	65.5 (58.7-75.5)	0.661
Creatinine, mmol/l	84.2 (72.8-98.2)	82.6 (71.5-94.8)	0.363	84.9 (73.4-98.2)	82.6 (71.6-94.9)	0.385
Glucose, mmol/l	6.4 (5.4-7.8)	6.6 (5.6-8.3)	0.140	6.7 (5.4-8.1)	7 (5.6-8.2)	0.708
AAT	25 (20-34)	26 (20-43)	0.361	27 (20-34)	35 (23.5-49.5)	0.030
ALT	24.5 (16-36)	27 (17-38)	0.611	25 (17-35)	29 (17-36)	0.610
Potassium, mmol/l	4.3 (3.8-4.7)	4.3 (3.9-4.7)	0.780	4.3±0.6	4.3±0.5	0.707

LVEF—left ventricular ejection fraction, LVESD—left ventricular end systolic diameter, LVEDD—left ventricular end diastolic diameter, LVESV—left ventricular end systolic volume, LVEDV—left ventricular end diastolic volume, WBC – white blood cells, AAT – aspartate aminotransferase, ALT – alanine aminotransferase.

Table S2. Dynamics of echocardiography parameters and laboratory data (Rosuvastatin vs. Control Group).

Parameter	Unmatched raw data			Propensity matched 1:1		
	Rosuvastatin (n=296)	No statins (n=498)	p	Rosuvastatin (n=223)	No statins (n=223)	p
Echocardiographic indicators initially						
LVEF, %	59 (55.7-62.1)	58 (54-62.4)	0.386	59 (55.5-62)	59 (55-62)	0.778
LVESD, cm	3.3 (3.1-3.6)	3.4 (3.2-3.8)	0.014	3.4 (3.2-3.7)	3.5 (3.2-3.8)	0.213
LVEDD, cm	5 (4.7-5.3)	5 (4.7-5.4)	0.010	5 (4.7-5.3)	5.1 (4.7-5.4)	0.118
LVESV, ml	47.2 (37.9-58.1)	50 (41-64)	0.005	47.4 (41-58.1)	50.9 (41-65)	0.035
LVEDV, ml	118.2 (102.4-135.3)	123 (105-141.3)	0.006	118.2 (102.4-135.3)	123.8 (107.5-145)	0.033
Echocardiographic indicators 2 day after operation						
LVEF, %	55.7 (53-57)	55.6 (53-57.1)	0.700	55.4 (52.8-57)	55 (53-57)	0.979
LVESV, ml	43 (35-50)	46 (38-55.5)	0.009	44.1 (35.5-52.5)	45.5 (38-55)	0.261
LVEDV, ml	97.3 (81-112)	101 (88.5-122)	<0.001	98 (83.3-114)	102.4 (89-124)	0.016
Fluid in the pericardium, n (%)	25 (8.4)	22 (4.4)	0.026	19 (8.5)	10 (4.5)	0.061
Fluid in the pleura, n (%)	46 (15.5)	38 (7.6)	0.002	36 (16.1)	22 (9.9)	0.031
Echocardiographic indicators 4 day after operation						
LVEF, %	55.9 (54-57)	56 (53.8-58)	0.577	55.9 (54.2-57.4)	56 (54.1-58)	0.704
LVESV, ml	43 (35-50.9)	44.1 (36-53)	0.221	44.1 (36-51)	43 (35-52)	0.709
LVEDV, ml	97 (82-113)	102 (86-120)	0.021	98.5 (84-115)	98.5 (83.1-118)	0.664
Fluid in the pericardium, n (%)	46 (15.5)	52 (10.4)	0.170	30 (13.5)	25 (11.2)	0.792
Fluid in the pleura, n (%)	37 (12.5)	48 (9.6)	0.628	29 (13)	25 (11.2)	0.815
Laboratory characteristics initially						
WBC, 10*9/l	7.7 (6.6-8.9)	7.7 (6.5-8.8)	0.800	7.7 (6.8-9)	7.5 (6.5-8.6)	0.125
Neutrophils, 10*9/l	4.6 (3.8-5.4)	4.5 (3.7-5.3)	0.564	4.7 (3.9-5.5)	4.5 (3.7-5.1)	0.088
Neutrophils, %	58.4 (52.2-64.6)	58 (53.4-63.6)	0.748	58.6 (52.4-65.2)	58.6 (54-64.4)	0.854
Creatinine, mmol/l	85.3 (74.5-99.7)	84.1 (73-97.6)	0.111	88 (75-99.9)	87 (75.1-101.1)	0.724
Glucose, mmol/l	5.5 (5-6.3)	5.4 (5-6.1)	0.035	5.5 (5-6.2)	5.5 (5.1-6.3)	0.779
AAT	22 (18.5-28.5)	22 (18-28)	0.666	22 (19-34)	22 (18-28)	0.236
ALT	26.5 (19-41.5)	24 (18-38)	0.251	29.5 (20-45.5)	22.5 (18-32.5)	0.022
Potassium, mmol/l	4.5 (4.3-4.9)	4.5 (4.2-4.8)	0.935	4.6 (4.3-4.9)	4.6 (4.3-4.8)	0.422
Laboratory characteristics 2 day after operation						
WBC, 10*9/l	13 (10.7-16.3)	13.1 (10.5-15.9)	0.441	12.8 (10.3-16.1)	12.7 (10.3-15.8)	0.505
Neutrophils, 10*9/l	11.2 (8.9-15.4)	10.9 (8.4-13.2)	0.091	10.8 (8.3-15.4)	10.8 (8.5-14.3)	0.671
Neutrophils, %	83.2 (76.7-87.4)	81 (71-86.6)	0.037	81.9 (73.9-87.3)	83.3 (75-86.2)	0.896
Creatinine, mmol/l	80.1 (68-94.7)	79 (69.6-94.3)	0.975	80.3 (68.9-94.3)	79 (71-98)	0.551
Glucose, mmol/l	7.6 (6.4-10)	7.4 (6.1-9.7)	0.177	7.4 (6.3-10)	7.5 (6.5-10)	0.686
AAT	31 (22-49)	29 (21-47)	0.214	32 (23.5-48)	28 (22-51.5)	0.289
ALT	22 (16-32)	20 (14-30)	0.350	24 (17-35)	19 (15-28.5)	0.019

Potassium, mmol/l	4.2 (3.9-4.5)	4.2 (3.9-4.5)	0.209	4.2 (3.9-4.6)	4.3 (3.9-4.5)	0.949
Laboratory characteristics 4 day after operation						
WBC, 10*9/l	11.3 (9.4-13.6)	10.7 (8.9-12.6)	0.019	11.3 (9.4-13.6)	10.8 (9.1-12.6)	0.059
Neutrophils, 10*9/l	7.5 (5.9-9.9)	6.7 (5.4-8.3)	0.002	7.5 (6.4-9.9)	6.7 (5.4-8.4)	0.010
Neutrophils, %	66 (59.8-75.2)	63.6 (57.7-70.2)	0.001	67.8±10.3	64.9±10.1	0.015
Creatinine, mmol/l	82.6 (71.5-94.8)	82.4 (72-96.7)	0.804	81.9 (71.6-94)	84.1 (73-98.2)	0.358
Glucose, mmol/l	6.6 (5.6-8.3)	6.5 (5.6-8.7)	0.775	6.6 (5.6-8.5)	6.5 (5.5-9)	0.729
AAT	26 (20-43)	25 (17-33.5)	0.105	25 (20.5-37.5)	21 (19-31)	0.149
ALT	27 (17-38)	24 (16.5-41.5)	0.464	26 (17-38.5)	23 (17-33)	0.313
Potassium, mmol/l	4.3 (3.9-4.7)	4.3 (3.9-4.7)	0.289	4.3±0.5	4.4±0.6	0.165

LVEF—left ventricular ejection fraction, LVESD—left ventricular end systolic diameter, LVEDD—left ventricular end diastolic diameter, LVESV—left ventricular end systolic volume, LVEDV—left ventricular end diastolic volume, WBC – white blood cells, AAT – aspartate aminotransferase, ALT – alanine aminotransferase.

Table S3. Dynamics of echocardiography parameters and laboratory data (Atorvastatin vs. Control Group).

Parameter	Unmatched raw data			Propensity matched 1:1		
	Atorvastatin (n=164)	No statins (n=498)	p	Atorvastatin (n=124)	No statins (n=124)	p
Echocardiographic indicators initially						
LVEF, %	59.5 (54.2-63)	58 (54-62.4)	0.353	59 (55.2-63)	58 (54-62)	0.105
LVESD, cm	3.4 (3.1-3.7)	3.4 (3.2-3.8)	0.411	3.4 (3.1-3.7)	3.5 (3.2-3.8)	0.312
LVEDD, cm	5 (4.7-5.4)	5 (4.7-5.4)	0.661	5.1 (4.7-5.4)	5.1 (4.8-5.4)	0.452
LVESV, ml	50.5 (39.5-60)	50 (41-64)	0.321	50.5 (39.5-58.6)	51 (42-66.5)	0.134
LVEDV, ml	123.8 (105.5-141.3)	123 (105-141.3)	0.926	123.8 (102.4-141.3)	125 (110-148)	0.292
Echocardiographic indicators 2 day after operation						
LVEF, %	55 (52-56)	55.6 (53-57.1)	0.017	55 (52-56)	55 (53.5-57)	0.069
LVESV, ml	41 (34-48)	46 (38-55.5)	0.002	42.5 (35-51)	46 (41-60)	0.010
LVEDV, ml	95 (77.5-114)	101 (88.5-122)	0.001	97 (80-116)	104.5 (88.5-125)	0.022
Fluid in the pericardium, n (%)	16 (9.8)	22 (4.4)	0.018	14 (11.3)	5 (4)	0.029
Fluid in the pleura, n (%)	25 (15.2)	38 (7.6)	<0.001	18 (14.5)	5 (4)	0.001
Echocardiographic indicators 4 day after operation						
LVEF, %	55 (53-56.7)	56 (53.8-58)	0.022	55 (53-57)	55 (54-57.5)	0.431
LVESV, ml	41 (34-50)	44.1 (36-53)	0.068	41 (34-48)	47.4 (39-58)	0.003
LVEDV, ml	97.5 (80.5-114)	102 (86-120)	0.031	98±22.4	108.4±24.3	0.003
Fluid in the pericardium, n (%)	40 (24.4)	52 (10.4)	<0.001	32 (25.8)	15 (12.1)	0.005
Fluid in the pleura, n (%)	37 (22.6)	48 (9.6)	<0.001	31 (25)	14 (11.3)	0.001
Laboratory characteristics initially						
WBC, 10*9/l	7.7 (6.6-9)	7.7 (6.5-8.8)	0.832	7.9 (6.7-9.3)	7.6 (6.6-8.7)	0.173
Neutrophils, 10*9/l	4.6 (3.9-5.6)	4.5 (3.7-5.3)	0.425	4.8 (3.9-6.1)	4.5 (4-5.1)	0.263
Neutrophils, %	58.7 (53-64.8)	58 (53.4-63.6)	0.712	60 (53.2-67)	58.4 (54.1-63.1)	0.811
Creatinine, mmol/l	86.3 (75.8-96.2)	84.1 (73-97.6)	0.471	85 (75.8-96)	83.4 (73.2-99.8)	0.850
Glucose, mmol/l	5.6 (5-6.4)	5.4 (5-6.1)	0.146	5.7 (4.9-6.3)	5.5 (5.1-6.1)	0.836
AAT	23 (18-38)	22 (18-28)	0.343	22.5 (18-38)	22 (18.8-26)	0.176
ALT	27 (18-40)	24 (18-38)	0.413	27 (19-38)	21.5 (15.5-32)	0.071
Potassium, mmol/l	4.4 (4.2-4.7)	4.5 (4.2-4.8)	0.147	4.4 (4.2-4.7)	4.5 (4.3-4.7)	0.529
Laboratory characteristics 2 day after operation						
WBC, 10*9/l	12.8 (10.5-15)	13.1 (10.5-15.9)	0.738	12.8 (10.6-15)	13.6 (10.4-16.5)	0.437
Neutrophils, 10*9/l	10.9 (8.8-13.7)	10.9 (8.4-13.2)	0.620	10.8 (8.8-13.7)	12.1 (8.4-14.3)	0.646
Neutrophils, %	83.4 (76.9-86.7)	81 (71-86.6)	0.042	83.3 (76.4-87.2)	84 (76.9-86)	0.694
Creatinine, mmol/l	80.2 (69.1-97.9)	79 (69.6-94.3)	0.854	80 (69.6-95)	82.2 (72.2-103.5)	0.141
Glucose, mmol/l	7.3 (6-9.3)	7.4 (6.1-9.7)	0.358	7.6 (5.9-9.5)	7.7 (6.5-10.1)	0.132
AAT	33 (23.5-60.5)	29 (21-47)	0.021	35 (24-62)	30.5 (20-45)	0.086
ALT	24 (16.5-37.5)	20 (14-30)	0.033	24 (17-37)	19.5 (15-30)	0.113

Potassium, mmol/l	4.1 (3.8-4.5)	4.2 (3.9-4.5)	0.014	4.1 (3.8-4.4)	4.2 (3.9-4.5)	0.132
Laboratory characteristics 4 day after operation						
WBC, 10*9/l	10.5 (9-13.2)	10.7 (8.9-12.6)	0.876	10.1 (9-13)	11.2 (9.1-13.3)	0.202
Neutrophils, 10*9/l	7.8 (6.2-10)	6.7 (5.4-8.3)	0.002	7.5 (6.2-9.8)	8.1 (6.6-9.6)	0.802
Neutrophils, %	65.7 (60.3-71.9)	63.6 (57.7-70.2)	0.019	65.6±10.5	66.9±10	0.381
Creatinine, mmol/l	84.2 (72.8-98.2)	82.4 (72-96.7)	0.482	82.7 (72-96)	83.6 (72-98.2)	0.719
Glucose, mmol/l	6.4 (5.4-7.8)	6.5 (5.6-8.7)	0.169	6.3 (5.3-7.6)	6.4 (5.4-8.9)	0.270
AAT	25 (20-34)	25 (17-33.5)	0.425	24 (19-37)	21 (17-29)	0.156
ALT	24.5 (16-36)	24 (16.5-41.5)	0.721	25.5 (19.5-44)	19 (13-35)	0.129
Potassium, mmol/l	4.3 (3.8-4.7)	4.3 (3.9-4.7)	0.574	4.3 (3.9-4.7)	4.3 (3.9-4.7)	0.725

LVEF—left ventricular ejection fraction, LVESD—left ventricular end systolic diameter, LVEDD—left ventricular end diastolic diameter, LVESV—left ventricular end systolic volume, LVEDV—left ventricular end diastolic volume, WBC – white blood cells, AAT – aspartate aminotransferase, ALT – alanine aminotransferase.

Table S4. Medicamentous therapy (Atorvastatin vs. Rosuvastatin).

Parameter	Unmatched raw data			Propensity matched 1:1		
	Atorvastatin (n=164)	Rosuvastatin (n=296)	p	Atorvastatin (n=108)	No statins (n=108)	p
Drug therapy initially						
Beta-blockers, n (%)	140 (85.4)	237 (80.1)	0.157	88 (81.5)	91 (84.3)	0.588
ACE inhibitors, n (%)	109 (66.5)	177 (59.8)	0.158	77 (71.3)	72 (66.7)	0.462
Calcium antagonists, n (%)	44 (26.8)	61 (20.6)	0.128	26 (24.1)	26 (24.1)	1.000
NSAIDs, n (%)	7 (4.3)	75 (25.3)	<0.001	5 (4.6)	7 (6.5)	0.768
Drug therapy 1 day after operation						
Beta-blockers, n (%)	130 (79.3)	253 (85.5)	0.088	86 (79.6)	92 (85.2)	0.284
ACE inhibitors, n (%)	104 (63.4)	209 (70.6)	0.113	72 (66.7)	75 (69.4)	0.662
Calcium antagonists, n (%)	27 (16.5)	33 (11.1)	0.105	17 (15.7)	14 (13)	0.560
NSAIDs, n (%)	43 (26.2)	173 (58.4)	<0.001	30 (27.8)	29 (26.9)	0.879
Antiarrhythmic drugs, n (%)	11 (6.7)	26 (8.8)	0.443	7 (6.5)	7 (6.5)	1.000
Steroids, n (%)	136 (82.9)	230 (77.7)	0.183	89 (82.4)	83 (76.9)	0.311
Drug therapy 2 day after operation						
Beta-blockers, n (%)	135 (82.3)	258 (87.2)	0.262	87 (80.6)	91 (84.3)	0.567
ACE inhibitors, n (%)	99 (60.4)	208 (70.3)	0.038	72 (66.7)	72 (66.7)	1.000
Calcium antagonists, n (%)	28 (17.1)	35 (11.8)	0.114	20 (18.5)	14 (13)	0.262
NSAIDs, n (%)	58 (35.4)	189 (63.9)	<0.001	41 (38)	43 (39.8)	0.780
Antiarrhythmic drugs, n (%)	22 (13.4)	50 (16.9)	0.339	17 (15.7)	15 (13.9)	0.702
Steroids, n (%)	30 (18.3)	55 (18.6)	0.963	21 (19.4)	25 (23.1)	0.506
Drug therapy 4 day after operation						
Beta-blockers, n (%)	128 (78)	251 (84.8)	0.099	82 (75.9)	91 (84.3)	0.200
ACE inhibitors, n (%)	100 (61)	210 (70.9)	0.038	72 (66.7)	73 (67.6)	0.959
Calcium antagonists, n (%)	27 (16.5)	32 (10.8)	0.076	18 (16.7)	14 (13)	0.410
NSAIDs, n (%)	69 (42.1)	178 (60.1)	<0.001	47 (43.5)	39 (36.1)	0.220
Antiarrhythmic drugs, n (%)	24 (14.6)	45 (15.2)	0.900	18 (16.7)	14 (13)	0.410
Steroids, n (%)	12 (7.3)	20 (6.8)	0.801	8 (7.4)	9 (8.3)	1.000

ACE – angiotensin-converting-enzyme, NSAIDs – nonsteroidal anti-inflammatory drugs.

Table S5. Medicamentous therapy (Rosuvastatin vs. Control Group).

Parameter	Unmatched raw data			Propensity matched 1:1		
	Rosuvastatin (n=296)	No statins (n=498)	p	Rosuvastatin (n=223)	No statins (n=223)	p
Drug therapy initially						
Beta-blockers, n (%)	237 (80.1)	324 (65.1)	<0.001	170 (76.2)	168 (75.3)	0.891
ACE inhibitors, n (%)	177 (59.8)	258 (51.8)	0.029	124 (55.6)	121 (54.3)	0.775
Calcium antagonists, n (%)	61 (20.6)	93 (18.7)	0.505	48 (21.5)	49 (22)	0.909
NSAIDs, n (%)	75 (25.3)	168 (33.7)	0.013	69 (30.9)	72 (32.3)	0.760
Drug therapy 1 day after operation						
Beta-blockers, n (%)	253 (85.5)	386 (77.5)	0.007	188 (84.3)	183 (82.1)	0.527
ACE inhibitors, n (%)	209 (70.6)	290 (58.2)	0.001	156 (70)	161 (72.2)	0.602
Calcium antagonists, n (%)	33 (11.1)	50 (10)	0.628	26 (11.7)	18 (8.1)	0.204
NSAIDs, n (%)	173 (58.4)	355 (71.3)	<0.001	143 (64.1)	153 (68.6)	0.316
Antiarrhythmic drugs, n (%)	26 (8.8)	40 (8)	0.717	16 (7.2)	17 (7.6)	0.856
Steroids, n (%)	230 (77.7)	405 (81.3)	0.197	173 (77.6)	179 (80.3)	0.486
Drug therapy 2 day after operation						
Beta-blockers, n (%)	258 (87.2)	402 (80.7)	0.022	194 (87)	189 (84.8)	0.497
ACE inhibitors, n (%)	208 (70.3)	298 (59.8)	0.003	157 (70.4)	162 (72.6)	0.600
Calcium antagonists, n (%)	35 (11.8)	56 (11.2)	0.799	25 (11.2)	22 (9.9)	0.644
NSAIDs, n (%)	189 (63.9)	396 (79.5)	<0.001	160 (71.7)	161 (72.2)	0.916
Antiarrhythmic drugs, n (%)	50 (16.9)	71 (14.3)	0.324	35 (15.7)	35 (15.7)	1.000
Steroids, n (%)	55 (18.6)	112 (22.5)	0.177	43 (19.3)	54 (24.2)	0.198
Drug therapy 4 day after operation						
Beta-blockers, n (%)	251 (84.8)	402 (80.7)	0.201	191 (85.7)	186 (83.4)	0.513
ACE inhibitors, n (%)	210 (70.9)	302 (60.6)	0.005	161 (72.2)	159 (71.3)	0.833
Calcium antagonists, n (%)	32 (10.8)	57 (11.4)	0.759	23 (10.3)	23 (10.3)	1.000
NSAIDs, n (%)	178 (60.1)	403 (80.9)	<0.001	158 (70.9)	158 (70.9)	1.000
Antiarrhythmic drugs, n (%)	45 (15.2)	69 (13.9)	0.627	33 (14.8)	37 (16.6)	0.603
Steroids, n (%)	20 (6.8)	38 (7.6)	0.629	15 (6.7)	21 (9.4)	0.297

ACE – angiotensin-converting-enzyme, NSAIDs – nonsteroidal anti-inflammatory drugs.

Table S6. Medicamentous therapy (Atorvastatin vs. Control Group).

Parameter	Unmatched raw data			Propensity matched 1:1		
	Atorvastatin (n=164)	No statins (n=498)	p	Atorvastatin (n=124)	No statins (n=124)	p
Drug therapy initially						
Beta-blockers, n (%)	140 (85.4)	324 (65.1)	<0.001	104 (83.9)	93 (75)	0.084
ACE inhibitors, n (%)	109 (66.5)	258 (51.8)	0.001	83 (66.9)	78 (62.9)	0.506
Calcium antagonists, n (%)	44 (26.8)	93 (18.7)	0.025	32 (25.8)	20 (16.1)	0.061
NSAIDs, n (%)	7 (4.3)	168 (33.7)	<0.001	5 (4)	3 (2.4)	0.722
Drug therapy 1 day after operation						
Beta-blockers, n (%)	130 (79.3)	386 (77.5)	0.667	97 (78.2)	95 (76.6)	0.761
ACE inhibitors, n (%)	104 (63.4)	290 (58.2)	0.252	81 (65.3)	73 (58.9)	0.295
Calcium antagonists, n (%)	27 (16.5)	50 (10)	0.027	22 (17.7)	16 (12.9)	0.290
NSAIDs, n (%)	43 (26.2)	355 (71.3)	<0.001	35 (28.2)	34 (27.4)	0.887
Antiarrhythmic drugs, n (%)	11 (6.7)	40 (8)	0.590	9 (7.3)	9 (7.3)	1.000
Steroids, n (%)	136 (82.9)	405 (81.3)	0.679	102 (82.3)	106 (85.5)	0.490
Drug therapy 2 day after operation						
Beta-blockers, n (%)	135 (82.3)	402 (80.7)	0.486	102 (82.3)	97 (78.2)	0.351
ACE inhibitors, n (%)	99 (60.4)	298 (59.8)	0.861	77 (62.1)	77 (62.1)	1.000
Calcium antagonists, n (%)	28 (17.1)	56 (11.2)	0.049	23 (18.5)	15 (12.1)	0.158
NSAIDs, n (%)	58 (35.4)	396 (79.5)	<0.001	48 (38.7)	43 (34.7)	0.510
Antiarrhythmic drugs, n (%)	22 (13.4)	71 (14.3)	0.802	15 (12.1)	24 (19.4)	0.116
Steroids, n (%)	30 (18.3)	112 (22.5)	0.256	23 (18.5)	40 (32.3)	0.013
Drug therapy 4 day after operation						
Beta-blockers, n (%)	128 (78)	402 (80.7)	0.477	98 (79)	98 (79)	0.896
ACE inhibitors, n (%)	100 (61)	302 (60.6)	0.915	78 (62.9)	76 (61.3)	0.856
Calcium antagonists, n (%)	27 (16.5)	57 (11.4)	0.092	22 (17.7)	16 (12.9)	0.302
NSAIDs, n (%)	69 (42.1)	403 (80.9)	<0.001	54 (43.5)	54 (43.5)	0.954
Antiarrhythmic drugs, n (%)	24 (14.6)	69 (13.9)	0.796	13 (10.5)	24 (19.4)	0.046
Steroids, n (%)	12 (7.3)	38 (7.6)	0.901	8 (6.5)	9 (7.3)	0.807

ACE – angiotensin-converting-enzyme, NSAIDs – nonsteroidal anti-inflammatory drugs.
